# The characteristics and health service utilization of adolescents with low back pain in a suburban pediatric health care system: analysis of health records data

**DOI:** 10.1186/s12998-025-00617-9

**Published:** 2025-11-28

**Authors:** Aubrianna L. Jones, Jeffrey A. King, Michael S. Swain, Katherine A. Pohlman, Channing Tassone, Robert J. Trager

**Affiliations:** 1 School of Health and Movement Sciences, Missouri Baptist University, One College Park Drive, St. Louis, MO 63141 USA; 2 Department of Neurosurgery, Medical College of Wisconsin, Milwaukee, WI USA; 3 Department of Chiropractic, Faculty of Medicine Health and Human Sciences, Macquarie University, Sydney, NSW Australia; 4 Parker University, Research Center, Dallas, TX USA; 5 Orthopaedic Surgery, Children’s Wisconsin, Milwaukee, WI USA; 6 Connor Whole Health, University Hospitals Cleveland Medical Center, Cleveland, OH USA; 7 Department of Family Medicine and Community Health, Case Western Reserve University School of Medicine, Cleveland, OH USA

**Keywords:** Low back pain, Pain management, Adolescent

## Abstract

**Background:**

Low back pain (LBP) is increasingly common among adolescents, yet little is known about the healthcare utilization in this population. We aimed to describe the characteristics and treatment patterns of adolescents with LBP presenting to a specialized comprehensive pediatric health system.

**Methods:**

This retrospective single-arm cohort design analyzed de-identified data from a suburban healthcare system. Using the TriNetX analytics platform, we queried electronic health records for adolescents aged 12–18 years with a new diagnosis of LBP between 2018 through 2022 without serious pathology such as cancer or infection. Key variables included baseline patient demographics, comorbidities, initial care setting, and the proportion and count of use of broad categories of healthcare services over a one-year follow-up window.

**Results:**

Our query identified 6,350 adolescents with LBP (mean age [standard deviation] of 14.8 [1.8] years; 60.6% female). The most common initial setting was ambulatory (80.5%). Services received by patients included non-opioid medication (38.8%), non-pharmacological conservative care (26.1%), diagnostic imaging (29.4%), opioids (11.3%), surgery (0.3%), and interventional injection therapies (≤ 0.2%).

**Conclusion:**

Among adolescents with newly diagnosed LBP from a specialized comprehensive pediatric healthcare system in Wisconsin from 2018 to 2022, 38.8% were prescribed non-opioid medications, 29.4% obtained diagnostic imaging, 26.1% had non-pharmacological conservative care, and 11.3% were prescribed opioids. Future studies should explore these findings in other care settings and examine optimal care pathways and associated clinical outcomes.

**Supplementary Information:**

The online version contains supplementary material available at 10.1186/s12998-025-00617-9.

## Introduction

Low back pain (LBP) is a leading cause of disability worldwide [1]. Research indicates that the incidence of LBP rises significantly during adolescence [2] with one study reporting that approximately 50% of adolescents internationally experience back pain on a monthly basis [3]. Additionally, the prevalence of LBP among adolescents has increased between 2001 and 2002 and 2013–2014, [3] further contributing to the growing burden on pediatric healthcare systems [4, 5]. In the United States, the mean annual healthcare cost for back pain in individuals aged 13–17 is estimated at $5,980 [6].

Low back pain is the most common musculoskeletal reason adolescents seek healthcare [7]. Those who do seek care often report high pain intensity, significant functional limitations, and concerns about their condition [7]. Research examining treatment pathways for adolescents with LBP remains limited. While prior studies have explored opioid prescribing patterns and imaging utilization in pediatric LBP, there is little real-world data describing the full spectrum of care received by this population, including potential non-opioid medications, other conservative treatments, and interventions. Understanding these treatment patterns is essential to further optimize the care delivered to this population.

Health outcomes established during adolescence influence long-term health outcomes and the risk of developing chronic diseases [8]. Persistent LBP during adolescence has been associated with a more than fourfold increased likelihood of persistent LBP [9]. A prior episode of back pain remains the most consistent predictor of future occurrences [10]. Despite this, there is limited research characterizing healthcare utilization and health services patterns among adolescents with LBP. Further evidence of these characteristics may serve as a crucial step in understanding the clinical course and outcomes of care.

Current guidelines recommend first-line treatment options for adolescent non-specific LBP, which include physical activity, therapeutic exercise, and manual therapy. These interventions prioritize conservative, non-pharmacological approaches aimed at improving function, reducing pain, and preventing chronicity [11–13]. While non-opioid and non-pharmacological management is recommended as current best practices for managing adolescent LBP, there remains minimal real-world data on the extent to which the recommendations are being implemented.

Considering the need to address the burden of adolescent LBP and inform policies related to health service utilization in this population, the present study aimed to describe healthcare utilization among adolescents with LBP who seek care based on patient and clinical care characteristics.

## Methods

### Study design

This retrospective observational single-cohort study was conducted using the clinical research collaboration platform TriNetX (TriNetX, LLC., Cambridge, MA, US). The study included adolescents meeting eligibility criteria spanning January 1, 2018, through December 31, 2022, to allow for one year of follow-up time, with the end of the data range extending to December 31, 2023. Our query date was August 5, 2024. An institutional review board exemption was issued for this study due to de-identified data, thereby waiving the requirement for patient or parental consent. The study identification number is PRO00051702.

### Data source and setting

TriNetX is a clinical research platform that integrates real-world data from electronic health records. The platform is compliant with the Health Insurance Portability and Accountability Act (HIPAA) and provides tools to enable cohort retrieval, characterization, and analysis, enabling secure queries of de-identified, aggregated patient data [14]. The platform has been previously validated in terms of its continuity, completeness, and representativeness of medication-related data [15, 16]. TriNetX harmonizes data, mapping clinical terminologies for consistency, and allows for queries using standardized nomenclatures like the International Classification of Diseases (ICD), RxNorm, and Current Procedural Terminology (CPT). Available data include demographics, diagnoses, treatments, laboratory tests, and other data [14]. To further support de-identification, any patient counts less than 10 are shown as 10.

We utilized TriNetX to analyze longitudinal data from a single suburban, academically affiliated healthcare organization in the United States, capturing 1,030,573 unique patients of all ages at the time of the query. The specialized comprehensive pediatric health system is uniquely positioned for studying adolescent LBP due to its integrated, interdisciplinary approach to spine care. It comprises two hospitals and provides comprehensive pediatric healthcare services across 40 ambulatory specialties. As a Level 1 trauma center, the system provides full-spectrum care, ranging from general pediatrics to subspecialized services. The care setting is best described as a comprehensive pediatric healthcare system, delivering care across primary, secondary, and tertiary settings.

Notably, spine care within the health system includes an interdisciplinary spine clinic, integrating orthopedics, neurology, neurosurgery, chiropractic, pain management, physical medicine and rehabilitation (PM&R), psychology, and physical and occupational therapy. In 2024, the medical center provided care to 127,000 unique pediatric patients, totaling 706,618 visits across outpatient specialty clinics. This level of multidisciplinary collaboration provides a robust model for pediatric spine care.

### Eligibility criteria

We included adolescents aged 12 to 18 years with a new low back-related diagnosis, representative of the first instance of any of a range of diagnoses considered. New LBP-related diagnosis was operationalized as the first recorded instance of any LBP-related ICD-10 code within the patient's electronic health record in our healthcare system. Each patient could only be counted once in our cohort, so that the unit of analysis was the patient rather than the care episode. While patients were eligible to have had symptoms preceding their first recorded diagnosis (as undiagnosed LBP could not be reliably assessed and ICD-10 codes do not specify exact symptom duration), the index date represents their first formal diagnosis and entry into our healthcare system for LBP. Given the variability in diagnostic codes used for common low back-related complaints, we queried the data for a range of conditions, for instance, dorsalgia, spondylosis, and other common disorders. A detailed outline of the specific ICD-10 codes used for our inclusion criteria is available in the Additional file 1 (Table S1).

We excluded individuals with serious pathologies, such as infections, tumors, and inflammatory conditions occurring between two years before or after the index date (i.e., the date at which patients presented with LBP and met inclusionary criteria), to align with our methods of analyzing spinal conditions that could be treated conservatively. A detailed outline of the specific ICD-10 codes used for our exclusion criteria is available in the Additional file 1 (Table S2). Additionally, we excluded individuals with scoliosis as these patients represent a distinct clinical population with potentially different care pathways, greater resource utilization, and potential surgical interventions that would not be representative of conservative management approaches for less complex adolescent LBP [17, 18].

### Variables

Health service utilization was categorized into groups representing conservative non-pharmacological care, interventional care, surgical care, non-opioid medication, and opioid medication. We used a one-year follow-up window to explore care pathways after the index date. The services identified were not mutually exclusive, and adolescents were eligible to have multiple treatments during the follow-up window. Conservative care in this population was defined based on current guidelines as non-pharmacological care, including education, exercise instruction, manual therapy, chiropractic spinal manipulation, and passive modalities. Interventional care was defined as injection procedures to treat lumbar spine-related conditions. Surgical care was defined as surgeries aimed at treating lumbar spine-related conditions. Pharmacological treatment was subdivided into non-opioid and opioid medications. A detailed breakdown of CPT codes and medications used to define each care group can be found in the Additional file 1 (Table S3−S8).

### Statistical methods

The TriNetX online analytics platform was used to generate descriptive statistics, including proportions, means, and medians. De-identified, aggregated data were exported and plotted using R [19] and RStudio (version 4.2.2, Vienna, AT) and the ggplot2 [20] package to illustrate the proportion of adolescents with LBP receiving various healthcare services, including 95% confidence intervals (CIs) for each proportion. Follow-up (retention) was plotted using locally estimated scatterplot smoothing. There was not a priori sample size target, as the authors desired to attain and analyze the maximum available sample from the study data range.

## Results

### Patients

**Fig. 1:**
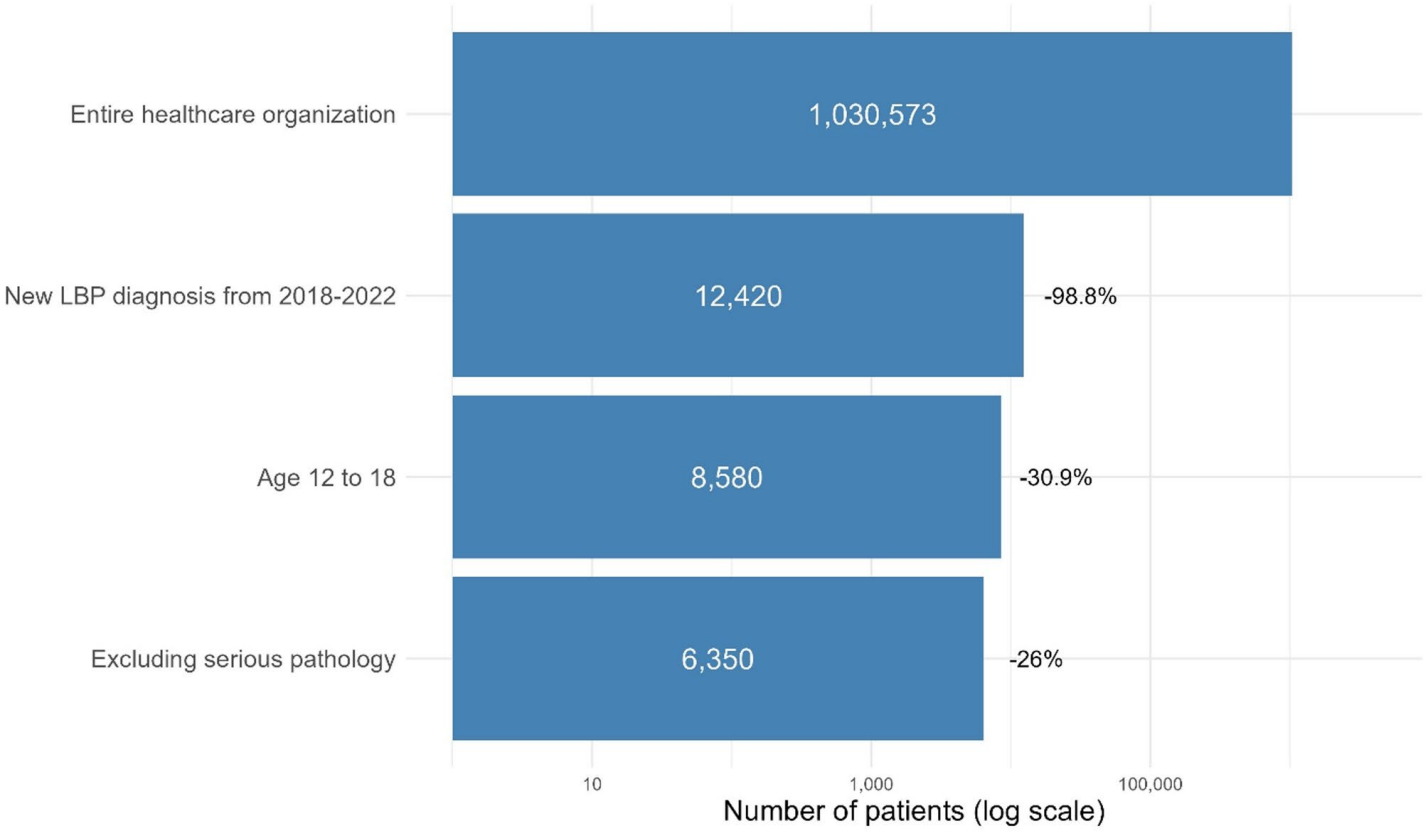
Funnel chart showing the sequential trimming of patients according to our selection criteria. The chart illustrates the number of patients at each stage, with the percentage of patients trimmed at each step but the first. The y-axis incorporates a logarithmic scale to enhance comparability of differences in the latter stages of selection. Abbreviations: low back pain (LBP)

Our query identified 6,350 adolescents with LBP meeting inclusion criteria, with a mean age (standard deviation, SD) of 14.8 (1.8) years, with 60.6% of patients being female. A funnel chart illustrating the effects of eligibility criteria on sample size is shown in Fig. 1. Most adolescents identified as white (68.7%). Additional demographics, characteristics, and co-morbidities of the study population are available in Table 1. The duration of follow-up was a mean of 275.8 days (SD = 142.4) and a median of 365 days (interquartile range = 0), with 77% of patients having at least the maximum follow-up duration. The follow-up duration metrics can be seen in Fig. 4.

**Table 1:** Patient Characteristics

Demographics	Mean (SD) or n (%)
Age at index date	14.8 (1.8)
Age at the time of query (y)	19.1 (2.5)
*Sex*	
Female	3850 (61)
Male	2500 (39)
*Ethnicity*	
Hispanic or Latino	920 (14)
Not Hispanic or Latino	5200 (82)
Unknown Ethnicity	240 (4)
*Race*	
Black or African American	1310 (21)
White	4360 (69)
Asian	110 (2)
American Indian or Alaska Native	20 (< 1)
Native Hawaiian or Other Pacific Islander	20 (< 1)
Other Race	0
Unknown Race	550 (9)
*Comorbidities*	
Chronic pain, not elsewhere classified	2140 (34)
Anxiety, dissociative, stress-related, somatoform and other nonpsychotic mental disorders	1170 (18)
Mood disorders	730 (12)
Overweight, obesity and other hyperalimentation	380 (6)
Persons with potential health hazards related to socioeconomic and psychosocial circumstances	250 (4)
Diabetes mellitus	90 (1)

### Key outcomes

**Fig. 2:**
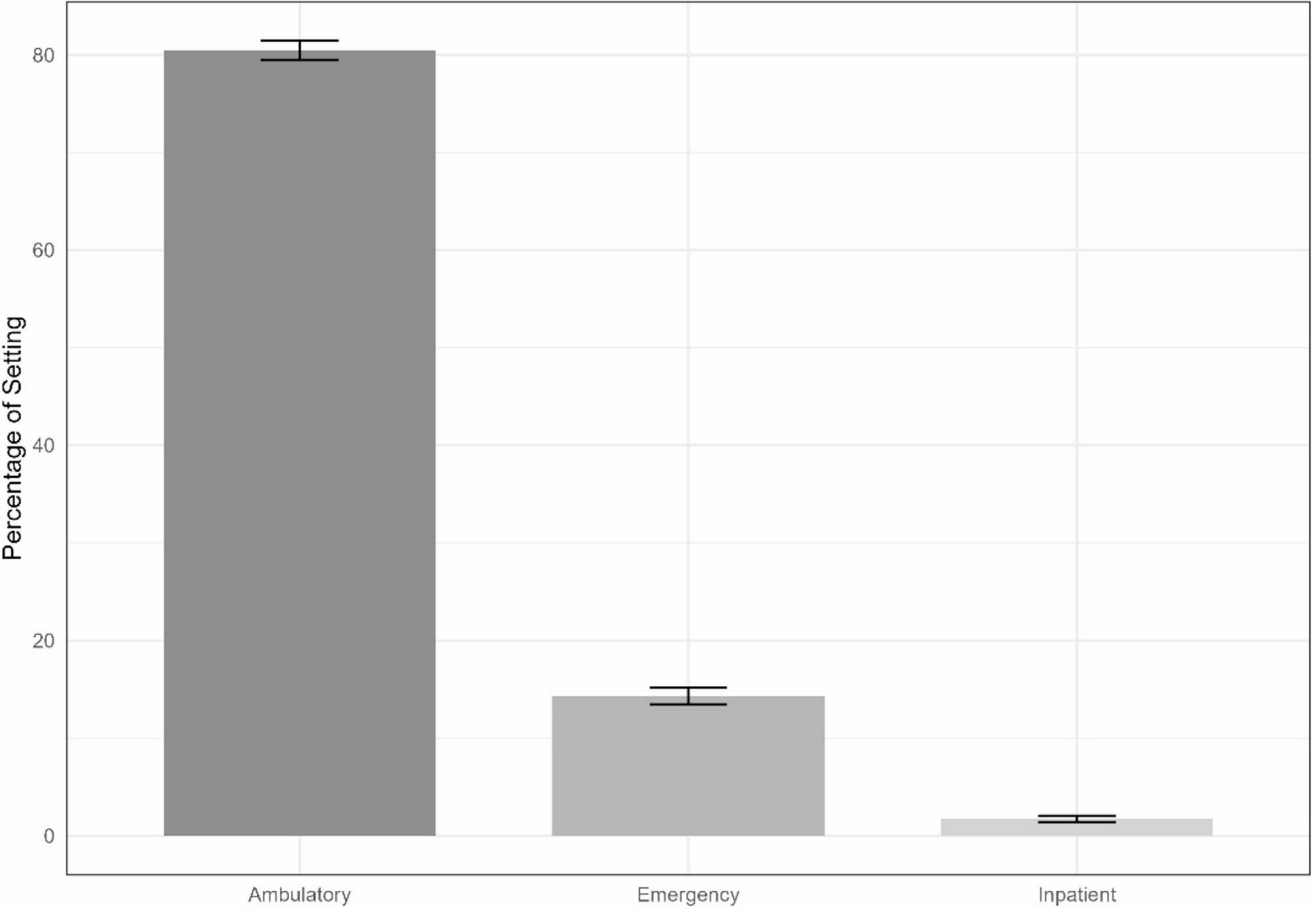
Proportion of initial care setting for adolescents with low back pain (*n* = 6,350)

The most common initial diagnosis location was an ambulatory setting (80.5%), followed by emergency department (14.3%) and inpatient (1.7%) settings (Fig. 2). The most prevalent diagnosis was *low back pain* (56%), followed by *dorsalgia unspecified* (30%) and *sacrococcygeal disorders, not elsewhere classified* (5%). See the Additional file 1 for a full list of diagnoses (Table S1).

**Fig. 3:**
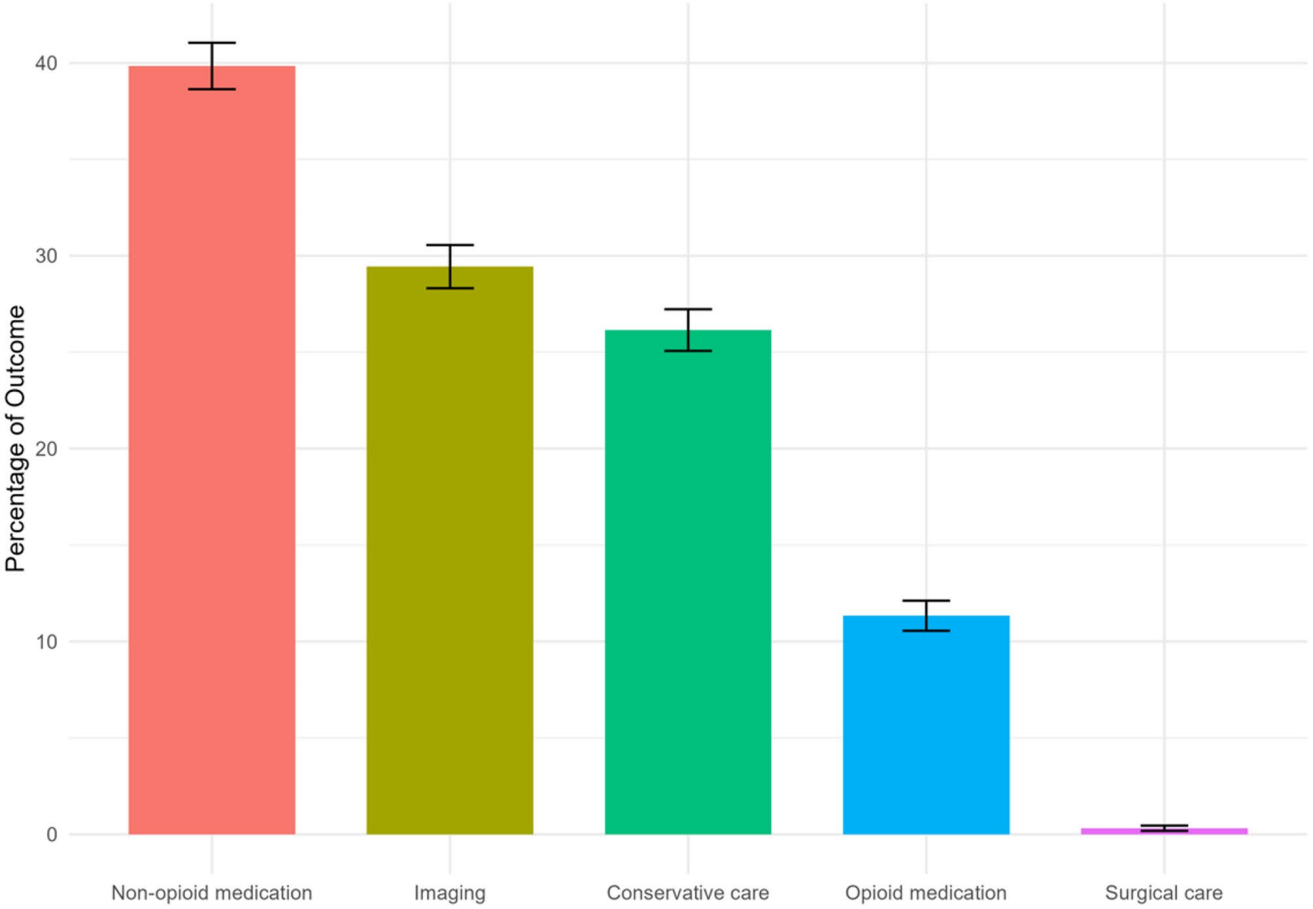
Proportion of broad service lines used by pediatric patients (*n* = 6,350). Note that interventions are not shown considering there were ≤ 10 instances of this service line and the exact amount cannot be shown for de-identification purposes

**Fig. 4:**
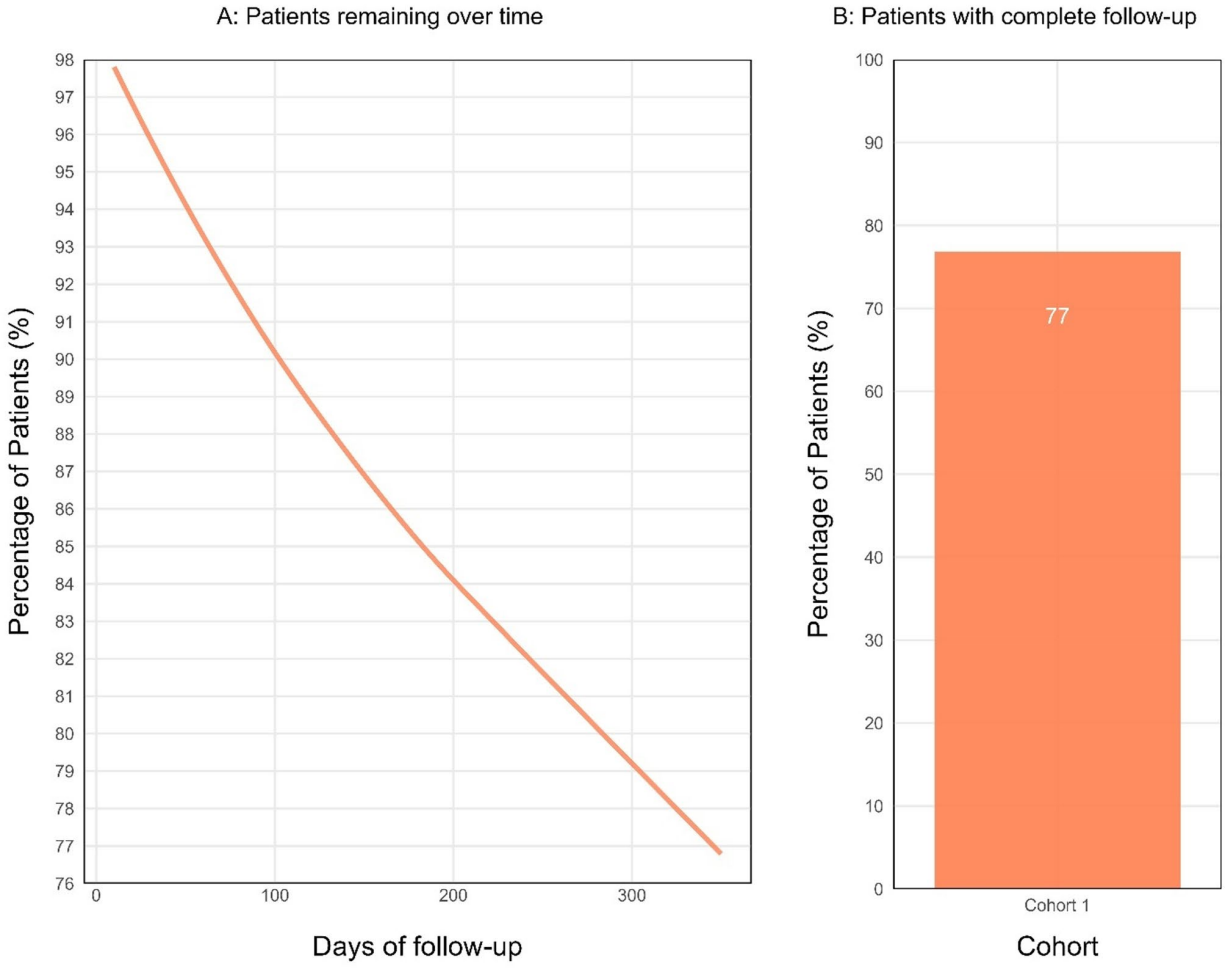
Follow-up data. **A**: This plot shows the percentage of patients remaining in the cohort throughout the duration of the one-year (365 day) follow-up. **B**: The bar plot displays the percentage of patients who remained in the cohort for at least the maximum follow-up time available (i.e., 77% of patients)

Services received by patients included non-opioid medication (38.8%), non-pharmacological conservative care (26.1%), diagnostic imaging (29.4%), opioids (11.3%), surgery (0.3%), and interventional therapies (≤ 0.2%) (Fig. 3). Additional details regarding the mean and median counts of separate instances of each service are available in Table 2. Comorbidities included chronic pain (34%), anxiety and related disorders (18%), mood disorders (12%), overweight/obesity (6%), psychosocial health hazards (4%), and diabetes mellitus (1%) (Fig. 4).

**Table 2:** Use of health services by pediatric patients (*N* = 6,350). Note: Total patient counts less than 10 are rounded up to 10 for de-identification purposes

Category	Patients n (%)	Mean count (SD)	Median count
Non-opioid medication	2,530 (39.8)	3.3 (7.6)	1
Conservative care	1,660 (26.1)	7.1 (6.2)	6
Opioid	720 (11.3)	5.2 (12.9)	1
Surgery	20 (0.3)	1.1 (0.3)	1
Intervention	≤ 10 (≤ 0.2)	1 (0)	1
Imaging	1,869 (29)	1.3 (0.6)	1

## Discussion

This study from a multi-level healthcare setting provides evidence from 6,350 care-seeking adolescents presenting with LBP. We found that care provided primarily included non-opioid medication and non-pharmacological conservative care. These findings may be reflective of the implementation of adult clinical practice guidelines. While similar, adolescent guidelines [11] advise against pharmacological treatment of recurrent or chronic non-specific back pain in adolescents [12, 13]. Additionally, we found that diagnostic imaging (29.4%) occurred more frequently than non-pharmacological conservative care (26.1%). Surgical care and interventional care, in this study, were uncommon, which may be explained by the fact that most cases of LBP in the adolescent population are seldom severe per progressive neurological deficit. The relatively high prevalence of chronic pain (34% based on ICD-10 coding) in our clinical population highlights that a substantial proportion of adolescents in the study presented with persistent or recurrent symptoms at their initial visit.

Our identified incidence of 11.3% for opioid prescription was expected, given the time-dependent decline in opioid utilization for pediatric LBP [21]. Illustratively, one nationally representative US study reported gradual reductions in opioid prescription among pediatric patients between 2008 and 2015. The most recent data from the study from 2015 reported an incidence of 7.7% for opioid prescriptions among patients aged 10 to 14, and an incidence of 20.9% among those aged 15 to 18 [22]. Accordingly, our study institution treatment patterns may reflect broader guideline-concordant trends to minimize opioid exposure among pediatric patients with LBP [11].

The negative impacts of LBP, particularly chronic LBP in adolescents, extend across several domains and include care-seeking behavior [23]. Population-level epidemiological studies have identified female sex and poor psychological health associated with chronic and impactful LBP in adolescents [24, 25]. In our study, approximately 1 in 5 adolescents with LBP had coexisting psychological health complaints. Clinical frameworks for the management of LBP in adolescents recommend the identification and targeting of psychological factors within the care pathway [11, 25]. Notwithstanding, direct comparisons of comorbidities in our study population and findings to existing literature should be made cautiously due to differences in study setting and patient characteristics. Our study was conducted within a large pediatric healthcare system that provides comprehensive care across primary, secondary, and tertiary settings. One study that included US data from 2005 to 2014 reported a weighted prevalence of diabetes of 0.8% among adolescents aged 12 to 19 [26], whereas our observed value was similar at 1.4%. Our observed proportion of those who were overweight or obese of 5.9% was lower than that of a previously-reported value for US adolescents, which of 21% [27], potentially reflective of regional or population-specific variance in our institution or underestimation of obesity diagnoses. While there is evidence that specific comorbidities such as obesity are associated with a higher risk of persistent LBP in adolescents [28], there remain knowledge gaps for comorbid conditions like diabetes [29]. Current guidelines do not address the management of comorbid conditions in adolescent LBP patients, highlighting a need for future research in this area.

The emphasis of conservative management in our study may reflect a predominance of non-urgent LBP diagnoses, aligning with recommendations for minimizing invasive procedures in adolescents LBP. Current diagnostic algorithms for adolescent LBP tend to recommend diagnostic imaging utilization. Notwithstanding, we observed a lower imaging rate than reported previously among adolescent LBP in the US (i.e., 29.4% vs. 37.8%) [30], but higher than rates in adults (24.8%) [31]. Jenkins et al. [32], highlights the problem of over imaging, which has challenged the adult LBP field. Diagnostic recommendations support imaging (namely MRI) for adolescent-specific LBP causes [11], although it remains unclear if the causes are similar to adults. Current guidelines for imaging adolescent LBP are derived from consensus due to sparse empirical evidence. In our study, comparative differences in imaging rates, while relatively small, may relate to institution-specific guidelines or other institution-specific characteristics.

Our observed frequency of chronic pain of 34% appears slightly higher than a previously-reported estimate of 20.8% (95% CI 19.2–22.4%) from a meta-analysis [33]. Our higher estimate may reflect care-seeking in a specialized multidisciplinary center, where chronic cases are more likely to present for care, rather than a true population rate. In general, comparison of the frequency of chronic pain in our setting versus others, or its impact on utilization should be interpreted cautiously, especially given our inability to analyze subgroups given the reliance upon aggregate data.

The present study is strengthened by having a low incidence of inpatient diagnoses and interventional procedures, which supports the validity of excluding serious pathology. Several limitations should be noted. This study lacks granular details on specific types of non-opioid medications, non-pharmacological conservative care, diagnostic imaging utilized, the specific healthcare professionals involved in patient care, patient-reported measures of pain severity, and healthcare costs. Data obtained from the TriNetX platform are provided in aggregate form with only pre-specified query variables available, while line-item (patient-level) data are not accessible per privacy regulations, preventing further inspection for granular treatment patterns. Given that recommendations and evidence vary for different approaches (e.g., evidence for exercise versus passive modalities differs for management of LBP in adolescents), and that care differs significantly between provider types (e.g., physical therapist versus neurologist), future studies should examine specific treatment components and provider characteristics to better understand optimal care pathways for adolescents with LBP. Selection bias is a potential concern, as only adolescents who sought care were included, thereby potentially underrepresenting those with mild or self-limiting symptoms. Some patients may have received care at outside institutions that were not linked to the dataset. Accordingly, comprehensive detail on co-management could have been missed. This study was conducted in a single multi-level pediatric academic medical center, so comparisons within the broader US adolescent population were not uniform or representative. The inclusion of specialized tertiary care services in our study likely increases the utilization rates. Therefore, our findings may not be generalizable to other healthcare settings, such as private primary care practices or international health systems, with different care pathways for adolescent LBP.

Given the increasing burden of adolescent LBP, further research is needed to understand healthcare utilization patterns to optimize policy and practice related to diagnostic tests and interventions. This study may serve as a model for replication to expand understanding in other healthcare settings. Additional emphasis should be placed on understanding co-management models of care. Future research should include clinical outcomes, such as pain, function, disability, and quality of life to better understand the effectiveness of interventions and the long-term impact of adolescent LBP on overall health.

## Conclusion

Among adolescents with newly diagnosed LBP from a specialized comprehensive pediatric healthcare system in Wisconsin from 2018 to 2022, 38.8% were prescribed non-opioid medications, 29.4% obtained diagnostic imaging, 26.1% had non-pharmacological conservative care, and 11.3% were prescribed opioids. These findings suggest guideline adherence and implementation of non-invasive management. Considering our results are specific to one healthcare institution, additional studies are needed to corroborate these findings in other settings and regions to further examine care patterns across diverse pediatric populations.

## Supplementary Information

Below is the link to the electronic supplementary material.

Supplementary Material 1

## Data Availability

All data generated or analyzed during this study are included in this published article and its Additional information file. Individuals interested in acquiring access to TriNetX may contact this organization (https://trinetx.com/).

## Abbreviations

CPT: Current Procedural Terminology
HIPAA: Health and Insurance Portability and Accountability Act
ICD: International Classification of Diseases
LBP: Low back pain
PM&R: Physical medicine and rehabilitation
SD: Standard deviation
