# Supplementary material for: The characteristics and health service utilization of adolescents with low back pain in a suburban pediatric health care system: analysis of health records data
Source: Chiropr Man Therap. 2025 Nov 28;33:54. doi: 10.1186/s12998-025-00617-9 (PMC12661819; doi:10.1186/s12998-025-00617-9)
Supplement: Supplementary file 1 — Supplementary Material 1 [file 12998_2025_617_MOESM1_ESM.docx]

*Table S1. Inclusion Diagnoses*

| ***Inclusion Diagnoses*** |  |  |  |
| --- | --- | --- | --- |
| **Name** | **ICD-10** | **Total N** | **Percentage** |
| Dorsalgia Unspecified | M54.9 | 1930 | 30% |
| Lesion of the sciatic nerve | G57.0 | 20 | 0% |
| Postural kyphosis, thoracolumbar region | M40.05 | <10 | 0 |
| Other secondary kyphosis, thoracolumbar region | M40.15 | <10 | 0 |
| Flatback syndrome, lumbar region | M40.36 | 0 | 0 |
| Postural lordosis, thoracolumbar region | M40.45 | 0 | 0 |
| Postural lordosis, lumbar region | M40.46 | <10 | 0 |
| Postural lordosis, lumbosacral region | M40.47 | 0 | 0 |
| Lordosis, unspecified, thoracolumbar region | M40.55 | <10 | 0 |
| Lordosis, unspecified, lumbar region | M40.56 | 20 | 0 |
| Lordosis, unspecified, lumbosacral region | M40.57 | 0 | 0 |
| Spondylolysis, thoracolumbar region | M43.05 | 0 |  |
| Spondylolysis, lumbar region | M43.06 | 210 | 3 |
| Spondylosis, lumbosacral region | M43.07 | 40 | 1 |
| Spondylosis, sacral and sacrococcygeal region | M43.08 | 0 |  |
| Unspecified kyphosis, thoracolumbar region | M40.295 | <10 | 0 |
| Spondylolysis, sacral and sacrococcygeal region | M43.08 | 0 | 0 |
| Spondylolisthesis, thoracolumbar region | M43.15 | <10 | 0 |
| Spondylolisthesis, lumbar region | M43.16 | 60 | 1 |
| Spondylolisthesis, lumbosacral region | M43.17 | 130 | 2 |
| Spondylolisthesis, sacral and sacrococcygeal region | M43.18 | <10 | 0 |
| Sacroiliitis, not elsewhere classified | M46.1 | <10 | 0 |
| Other spondylosis with radiculopathy, thoracolumbar region | M47.25 | 0 | 0 |
| Other spondylosis with radiculopathy, lumbar region | M47.26 | 0 | 0 |
| Other spondylosis with radiculopathy, lumbosacral region | M47.27 | 0 | 0 |
| Spondylosis without myelopathy or radiculopathy, thoracolumbar region | M47.815 | 0 | 0 |
| Spondylosis without myelopathy or radiculopathy, lumbar region | M47.816 | 20 | 0 |
| Spondylosis without myelopathy or radiculopathy, lumbosacral region | M47.817 | <10 | 0 |
| Spondylosis without myelopathy or radiculopathy, sacral and sacrococcygeal | M47.818 | <10 | 0 |
| Other spondylosis, thoracolumbar region | M47.895 | 0 | 0 |
| Other spondylosis, lumbar region | M47.896 | <10 | 0 |
| Other spondylosis, lumbosacral region | M47.897 | 0 | 0 |
| Other spondylosis, sacral and sacrococcygeal region | M47.898 | 0 | 0 |
| Spinal stenosis thoracolumbar region | M48.05 | 0 | 0 |
| Spinal stenosis lumbar region | M48.06 | 20 | 0 |
| Spinal stenosis, lumbosacral region | M48.07 | <10 | 0 |
| Spinal stenosis, sacral and sacrococcygeal region | M48.08 | 0 | 0 |
| Unspecified thoracic, thoracolumbar and lumbosacral intervertebral disc disorder | M51.9 | 20 | 0 |
| Intervertebral disc disorders with radiculopathy, thoracolumbar region | M51.15 | 0 | 0 |
| Intervertebral disc disorders with radiculopathy, lumbar region | M51.16 | <10 | 0 |
| Intervertebral disc displacement, lumbosacral region | M51.17 | <10 | 0 |
| Other intervertebral disc displacement, thoracolumbar region | M51.25 | 0 | 0 |
| Other intervertebral disc displacement, lumbar region | M51.26 | 70 | 1 |
| Other intervertebral disc displcement lumbosacral region | M51.27 | 30 | 0 |
| Other intervertebral disc degeneration, thoracolumbar region | M51.35 | <10 | 0 |
| Other intervertebral disc degeneration, lumbar region | M51.36 | 40 | 1 |
| Other intervertebral disc degeneration, lumbosacral region | M51.37 | 20 | 0 |
| Schmorl's nodes, thoracolumbar region | M51.45 | <10 | 0 |
| Schmorl's nodes, lumbar region | M51.46 | 20 | 0 |
| Schmorl's nodes, lumbosacral region | M51.47 | <10 | 0 |
| Other intervertebral disc disorders, thoracolumbar region | M51.85 | 0 | 0 |
| Other intervertebral disc disorders, lumbar region | M51.86 | <10 | 0 |
| Other intervertebral disc disorders, lumbosacral region | M51.87 | <10 | 0 |
| Sacrococcygeal disorders, not elsewhere classified | M53.3 | 340 | 5 |
| Other specified dorsopathies, thoracolumbar region | M53.85 | 40 | 1 |
| Other specified dorsopathies, lumbar region | M53.86 | 30 | 0 |
| Other specified dorsopathies, lumbosacral region | M53.87 | 0 | 0 |
| Lumbago with sciatica | M54.4 | 220 | 3 |
| Radiculopathy, sacral and sacrococcygeal region | M54.18 | <10 | 0 |
| Sciatica | M54.3 | 30 | 0 |
| Radiculopathy, lumbosacral region | M54.17 | <10 | 0 |
| Radiculopathy, lumbar region | M54.16 | 30 | 0 |
| Radiuclopathy, thoracolunbar region | M54.15 | 0 | 0 |
| Other specified dorsopathies, sacral and sacrococcygeal region | M53.88 | <10 | 0 |
| Osseous and subluxation stenosis of intervertebral foramina of lumbar region | M99.63 | 0 | 0 |
| Intervertebral disc stenosis of neural canal of lumbar region | M99.53 | <10 | 0 |
| Segmental and somatic dysfunction of pelvic region | M99.05 | <10 | 0 |
| Segmental and somatic dysfunction of sacral region | M99.04 | 20 | 0 |
| Segmental and somaric dysfunction of lumbar region | M99.03 | 20 | 0 |
| Muscle spasm of back | M62.830 | 70 | 1 |
| Low back pain | M54.5 | 3560 | 56 |
| Other biomechanical lesions of pelvic region | M99.85 | 0 | 0 |
| Other biomechanical lesions of sacral region | M99.84 | 0 | 0 |
| Other biomechanical lesions of lumbar region | M99.83 | 0 | 0 |
| Connective tissue and disc stenosis of intervertebral foramina of lumbar region | M99.73 | 0 | 0 |
| Unspecified injury of muscle, fascia and tendon of pelvis | S39.003 | <10 | 0 |
| Unspecified injury of muscle, fascia and tendon of lower back | S39.002 | <10 | 0 |
| Unspecified injury of pelvis | S39.93 | 0 | 0 |
| Unspecified injury of lower back | S39.92 | 0 | 0 |
| Other specified injuries of pelvis | S39.83 | 20 | 0 |
| Other specified injuries of lower back | S39.82 | <10 | 0 |
| Sprain of unspecified parts of lumbar spine and pelvis | S33.9 | <10 | 0 |
| Sprain of other parts of lumbar spine and pelvis | S33.8 | <10 | 0 |
| Sprain of sacroiliac joint | S33.6 | <10 | 0 |
| Sprain of ligaments of lumbar spine | S33.5 | 20 | 0 |
| Traumatic rupture of lumbar intervertebral disc | S33.0 | <10 | 0 |
| Other injury of muscle, fascia and tendon of pelvis | S39.093 | <10 | 0 |
| Other injury of muscle, fascia and tendon of lower back | S39.092 | <10 | 0 |
| Strain of muscle, fascia and tendon of pelvis | S39.013 | <10 | 0 |
| Strain of muscle, fascia and tendon of lower back | S39.012 | 200 | 3 |

*Table S2. Exclusion Diagnoses.*

| ***Exclusion Diagnoses*** |  |  |  |
| --- | --- | --- | --- |
| **Name** | **ICD-10** | **Total N** | **Percentage** |
| Osteomyelitis | M86 | <10 | 0 |
| Malignant neoplasms of bone and articular cartilage | C40-C41 | <10 | 0 |
| Scoliosis | M41 | 70 | 1% |
| Infectious arthropathies | M00-M02 | <10 | 0 |
| Other inflammatory spondylopathies | M46 | <10 | 0 |

*Table S3. Conservative Care CPT Codes.*

| **Name** | **CPT Code** |
| --- | --- |
| Therapeutic exercise | 97110 |
| Therapeutic activities dynamic activities | 97530 |
| Neuromuscular re-education | 97112 |
| Manual therapy | 97140 |
| Chiropractic manipulative treatment 1-2 regions | 98940 |
| Chiropractic manipulative treatment 3-4 regions | 98941 |
| Chiropractic manipulative treatment extraspinal | 98943 |
| Application of a modality | 97035 |
| Physical therapy evaluations | 1029677 |
| Physical Medicine and Rehabilitation Modalities | 1013490 |

*Table S4. Interventional Care CPT codes.*

| **Name** | **CPT Code** |
| --- | --- |
| Injection(s) of diagnostic or therapeutic substance(s) epidural or subarachnoid; lumbar or sacral | 62311 |
| Injection(s) of diagnostic or therapeutic substance(s) interlaminar epidural or subarachnoid lumbar or sacral | 62322 |
| Injection(s) of diagnostic or therapeutic substance(s) including needle of catheter placement, interlaminar epidural or subarachnoid lumbar or sacral | 62323 |
| Injection(s), including indwelling catheter placement, interlaminar epidural of subarachnoid, lumbar or sacral with imaging guidance | 62327 |
| Injection procedure for sacroiliac joint anesthetic/steroid with guidance including arthography | 27096 |
| Injection(s) anesthetic agent(s) and.or steroid; transforaminal epidural, with imaging guidance, lumbar or sacral, single level | 64483 |
| Injections(s) diagnostic or therapeutic agent, paravertebral facet with image guidance lumbar or sacral;single level | 64493 |
| Injections(s) diagnostic or therapeutic agent, paravertebral facet with image guidance lumbar or sacral; second level | 64494 |
| Injection(s) diagnostic or therapeutic agent, paravertebral facet with image guidance lumbar or sacral; third and any additional levels | 64495 |

*Table S5. Surgical Care CPT codes.*

| **Name** | **CPT** |
| --- | --- |
| Laminectomy, facetectomy and foraminotomy of spinal cord, cauda equina and/or nerve root[s], single vertebral segment;lumbar | 63047 |
| Laminectomy with exploration and/or decompression of spinal cord and/or cauda equina, without facetectomy, foraminotomy or discectomy, 1 or 2 vertebral segments; lumbar, except for spondylolisthesis | 63005 |
| Laminectomy with exploration and/or decompression of spinal cord and/or cauda equina, without facetectomy, foraminotomy or discectomy, 1 or vertebral segments; sacral | 63011 |
| Laminectomy with removal of abnormal facets and/or pars inter-articularis with decompression of cauda equina and nerve roots for spondylolisthesis lumbar | 63012 |
| Laminectomy with exploration and/or decompression of spinal cord and/or cauda equina, without facetecomty, foraminotomy or discectomy more than 2 vertebral segments; lumbar | 63017 |
| Laminectomy for excision or evacutaion of intraspinal lesion other than neoplasm extradural;lumbar | 63267 |
| Laminotomy with decompression of nerve root(s) including partial facetectomy, foraminotomy and/or excision of herniated intervertebral disc; 1 interspace, lumbar | 63030 |
| Laminotomy with decompression of nerve root(s) including partial facetectomy, foraminotomy and/or excision of herniated intervertebral disc reexploration; 1 interspace, lumbar | 63042 |
| Laminotomy with decompression of nerve root(s) including partial facetectomy, foraminotomy and/or excision of herniated intervertebral disc, reexploration, single interspace, each additional lumbar interspace | 63044 |
| Transpedicular approach with decompression of spinal cord, equina and/or nerve root(s), single segment; lumbar | 63056 |
| Arthrodesis, anterior interbody technique, including minimal discectomy to prepare interspace (other than for decompression) lumbar | 22558 |
| Verterbral corpectomy, partial or complete, lateral extracavitary approach with decompression of spinal cord and/or nerve root(s), lumbar single segment | 63102 |
| Vertebral corpectomy, partial or complete, lateral extracaviraty approach with decompresison of spinal cord and/or nerve root(s); thoracic or lumbar, each additional segment | 63103 |
| Vertebral corpectomy, partial or complete, transperitoneal or retroperitoneal approach with decompression of spinal cord, cauda equina or nerve root(s), lower thoracic, lumbar, or sacral; single segment | 63090 |
| Arthrodesis, posterior or posterolateral technique, including minimal discectomy to prepar interspace (other than for decompression); lumbar | 22612 |
| Arthrodesis, lateral extracavitary technique, including laminectomy and/or discectomy to prepare interspace (other than for decompression); lumbar | 22533 |
| Arthrodesis, posterior interbody technique, including laminectomy and/or discectomy to prepare interspace (other than for decompression), single interspace, lumbar | 22630 |
| Arthrodesis, combined posterior or posterolateral technique with posterior interbody technique including laminectomy and/or discectomy sufficient to prepare interspace, single interspace lumbar | 22633 |
| Arthrodesis, posterior interbody technique, including laminectomy and/or discectomy to prepare interspace (other than for decompression), single interspace, lumbar; each additional interspace | 22632 |
| Arthrodesis, pre-sacral interbody technique, including disc space preparation discectomy, with posterior instrumentation, with image guidance, includes bone graft when performed, L5-S1 interspace | 22586 |
| Arthrodesis, sacroiliac joint, open, includes obtaining bone graft, including instrumentation, when performed | 27280 |
| Arthrodesis, sacroiliac joint, percutaneous, or minimally invasive, with image guidance, includes obtaining bone graft when performed and placemewnt of transfixing device | 27279 |
| Decompression procedure, percutaneous of nucleus pulposus of intervertebral disc, any method utilizing needle based technique to remove disc marterial under fluoroscopic imaging or other form or indirect visualization, with discography and/or epidural injection(s) at the treated level(s), with performed, single or multiple levels, lumbar | 62287 |
| Percutaneous laminotomy/laminectomy (interlaminar approach) for decompression of neural elements, any method, under indirect image guidance, single or multiple levels, unilatera or bilateral lumbar | 0275T |
| Pelvic fixation other than sacrum | 22848 |
| Laminectomny with release of thethered spinal cord, lumbar | 63200 |
| Laminotomy, with decompression of nerve root(s), including partial facetectomy, foraminotomy and/or excision of herniated intervertebral disc; each additional interspace, cervical or lumbar | 63035 |
| Laminectomy, facetectomy and foraminotomy , single vertebral segment; each additional verterbral segment, cervical, thoracic, or lumbar | 63048 |
| Transpedicular approach with decompression of spinal cord, equina and/or nerve root(s), single segment; each additional segment, thoracic or lumbar | 63057 |
| Arthrodesis, anterior interbody technique, including minimal discectomy to prepare interspace; each additional interspace | 22585 |
| Arthrodesis, lateral extracavitary technique including minimal discectomy to preparte interspace; thoracic or lumbar each additional vertebral segment | 22534 |
| Arthrodesis, posterior or posterolateral technique, single interspace; each additional interspace | 22614 |
| Vertebral corpectomy, partial or complete transperitoneal or retroperitoneal approach with decompression of spinal cord, cauda equina or nerve root(s), lower thoracic, lumbar, or sacral; each additional segment | 63091 |
| Vertebral corpectomy, partial or complete, combined thoracolumbar approach with decompression of spinal cord, cauda equina ornerve root(s), lower thoracic or lumbar; single segment | 63087 |
| Verterbral corpectomy, partial or complete, combined thoracolumbar approach with decompression of spinal cord, cauda equina or nerve root(s), lower thoracic or lumbar; each additional segment | 63088 |
| Posterior non-segmental instrumentation | 22840 |
| Posterior segmental instrumentation; 13 or more vertebral segments | 22844 |

*Table S6. Imaging CPT codes.*

| **Name** | **CPT Code** |
| --- | --- |
| Magnetic resonance imaging, spinal canal and contents, lumbar | 1010405 |
| Computed tomography, lumbar spine | 1010395 |
| Radiologic examination, spine, lumbosacral | 1010381 |

*Table S7. Non-opioid Medication CPT codes.*

| **Name** | **CPT Code** |
| --- | --- |
| Skeletal muscle relaxants | MS200 |
| Non-opioid analgesics | CN103 |
| Nonsalicylare NSAIs, antirheumatic | MS102 |

*Table S8. Opioid Medication CPT codes.*

| **Name** | **CPT Code** |
| --- | --- |
| Opioid analgesics | CN101 |
